# Supplementary material for: Cupriavidus metallidurans CH34 Possesses Aromatic Catabolic Versatility and Degrades Benzene in the Presence of Mercury and Cadmium
Source: Microorganisms. 2022 Feb 21;10(2):484. doi: 10.3390/microorganisms10020484 (PMC8879955; doi:10.3390/microorganisms10020484)
Supplement: Supplementary file 1 [file microorganisms-10-00484-s001.zip › microorganisms-1587111-supplementary/FigureS1.pdf]

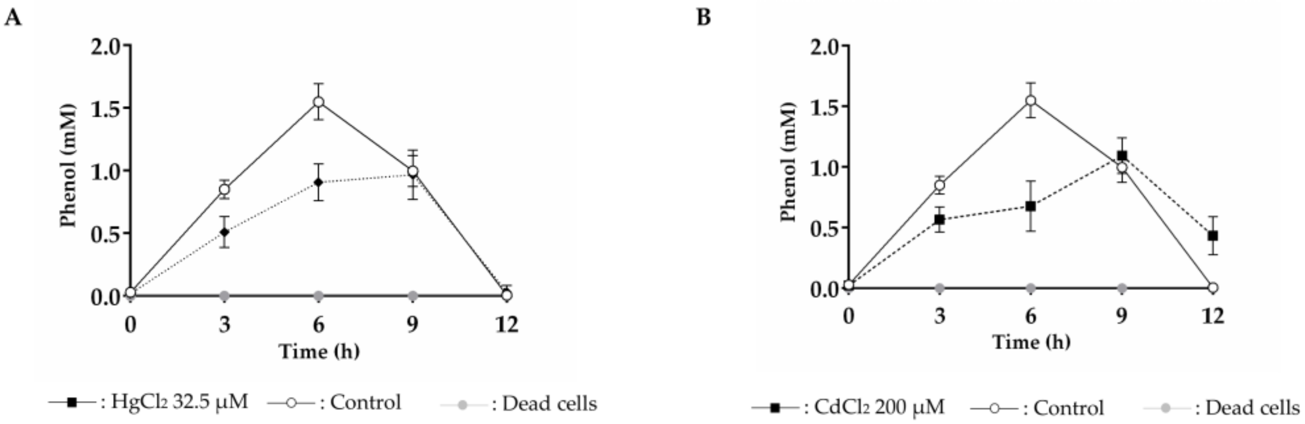

Figure S1. Effects of mercury and cadmium on phenol production in *C. metallidurans* CH34 during resting cell assays. (**A**) Effect of $HgCl_2$ 32.5 μM (**B**) Effect of $CdCl_2$ 200 μM. Complete depletion of phenol is observed at 12 h during control conditions. Each point is an average ± SDs of results from three independent resting cells assays.
