# Supplementary material for: Cupriavidus metallidurans CH34 Possesses Aromatic Catabolic Versatility and Degrades Benzene in the Presence of Mercury and Cadmium
Source: Microorganisms. 2022 Feb 21;10(2):484. doi: 10.3390/microorganisms10020484 (PMC8879955; doi:10.3390/microorganisms10020484)
Supplement: Supplementary file 1 [file microorganisms-10-00484-s001.zip › microorganisms-1587111-supplementary/Table S2.pdf]

Table S2. Primer sets designed and used in this study.

Gen	Name	Sequence (5'-3')	Efficiency (%)	Amplicon Size
tomA3	tomA3 Fw	TGATGCATTCGATGCCGAGT	106.8	219 pb
	tomA3 Rv	TGTCGGTGCCCAAGTCATTT		
tomB	tomB Fw	TTTTCCGCCACCCGATTCA	109.4	241 pb
	tomB Rv	AGACACAAATGCTCCCCGAA		
tmoA	tmoA Fw	TTCCAGCCAATCCCGTT	105.3	228 pb
	tmoA Rv	CATCGCAAGCAGTCGTTCAA		
phyC	phyC Fw	TCCTGTTGCTGCTTCCAGTAG	97.2	211 pb
	phyC Rv	TCCGGATGCCCAAGTATCAC		
catA1	catA1 Fw	TTGACCTGCCCTTCCATCAC	101.9	245 pb
	catA1 Rv	CTCAATTTCCTGGCCGAAGC		
catA2	catA2 Fw	CCTGCTTGGCAACTACTCGT	96.4	212 pb
	catA2 Rv	CGGCGGATACGAAGAAGTGA		
tomC	tomC Fw	CGCGGAGACAAAACGATCAA	99.5	250 pb
	tomC Rv	CATCAACGCCATCACCTTCAC		
tomD	tomD Fw	GGTGCCGGAAACATACTTGAAA	109.2	231 pb
	tomD Rv	CGAAAGAGTGCGCCGATTG		
rpoS	rpoS Fw	CGAGCGAGGTGGTATGTTCA	92.0	241 pb
	rpoS Rv	TATGCGACCTGGTGGATTCG		
benM	benM Fw	AGCACCTGATCGGCAAAACT	95.3	226 pb
	benM Rv	TCAAGGCACTCAAGGAAGGG		
catM	catM Fw	AAGCTTCATATGGCACAGCC	103.4	210 pb
	catM Rv	GAACCAGCGCTTTTCATTGC		
poxR	poxR Fw	CTCTGGCAGGACTCATGGGA	89.7	248 pb
	poxR Rv	CTGATGACGGAGTCGGGTTC		
tomR	tomR Fw	TCCCCCGTTTGCAAAACTCT	91.3	232 pb
	tomR Rv	ACAGAAGTTCGCGTGTAGGG		
gyrB	gyrB Fw	GATGGCTACAACGAGCAGGT	98.1	233 pb
	gyrB Rv	TTGGTCTGCGAGCTGAACTT		
