# Supplementary material for: Cupriavidus metallidurans CH34 Possesses Aromatic Catabolic Versatility and Degrades Benzene in the Presence of Mercury and Cadmium
Source: Microorganisms. 2022 Feb 21;10(2):484. doi: 10.3390/microorganisms10020484 (PMC8879955; doi:10.3390/microorganisms10020484)
Supplement: Supplementary file 1 [file microorganisms-10-00484-s001.zip › microorganisms-1587111-supplementary/TableS1.pdf]

Table S1. Bacterial multicomponent monooxygenases operon of *Cupriavidus metallidurans* strain CH34 and other Proteobacteria.

Classification by		Operon	BMMs	Organism	Location	Accession N°
Notomista et al., 2003	This study					
Group I	Clade IA	*phyZABCDE*	Phenol hydroxylase	*Cupriavidus metallidurans* CH34	C1	CP000352
		phlK1L1M1N1O1P1	Phenol hydroxylase	*Cupriavidus pinatubonensis* JMP134	C1	NC_007347
		dmpKLMNOPQ	Phenol hydroxylase	*Sphingobium phenoxybenzoativorans* SC_3	-	KX823580
		phyZABCDE	Phenol hydroxylase	*Ralstonia* sp. KN1	-	AB031996
	Clade IIB	*tbmABCDEF*	Toluene/benzene 2-monooxygenase	*Burkholderia cepacia* JS150	-	L40033
		tbc1ABCDEF	Phenol hydroxylase	*Burkholderia cepacia* JS150	-	AF282897
		phlK2L2M2N2O2P2	Phenol hydroxylase	*Cupriavidus pinatubonensis* JMP134	C2	NC_007348
		aphKLMNOP	Phenol hydroxylase	*Comamonas testosteroni* TA441	-	NZ_BKBW01000011
		tomA0A1A2A3A4A5	Toluene 2-monooxygenase	*Cupriavidus metallidurans* CH34	C1	CP000352
		tomA0A1A2A3A4A5	Toluene 2-monooxygenase	*Burkholderia vietnamensis* G4	pBVIE04	CP000620
	Clade IC	*dmpKLMNOP*	Phenol hydroxylase	*Alcaligenes aquatilis* QD168	C	CP032153
		dmpKLMNOP	Phenol hydroxylase	*Pseudomonas putida* CF600	pVI150	AB910524
		phhKLMNOP	Phenol hydroxylase	*Pseudomonas putida* P35X	-	X79063
Group II	-	*tbc2ABCDEF*	Toluene/benzene 2-monooxygenase	*Burkholderia cepacia* JS150	-	AF282898
		tbuA1UBVA2C	Toluene-3-monooxygenase	*Ralstonia pickettii* PKO1	-	AY541701
		tbcABCDEF	Toluene monooxygenase	*Cupriavidus pinatubonensis* JMP134	C2	NC_007348
		tmoABCDEF	Toluene monooxygenase	*Cupriavidus metallidurans* CH34	C1	CP000352
		tmoABCDEF	Toluene-4-monooxygenase	*Pseudomonas mendocina* KR1	-	AY552601
		tmoABCDEF	Toluene-3-monooxygenase	*Pseudomonas* sp. M4	-	KJ735680
