# Supplementary material for: Cupriavidus metallidurans CH34 Possesses Aromatic Catabolic Versatility and Degrades Benzene in the Presence of Mercury and Cadmium
Source: Microorganisms. 2022 Feb 21;10(2):484. doi: 10.3390/microorganisms10020484 (PMC8879955; doi:10.3390/microorganisms10020484)
Supplement: Supplementary file 1 [file microorganisms-10-00484-s001.zip › microorganisms-1587111-supplementary/TableS3.pdf]

Table S3. Predicted aromatic central pathways in *C. metallidurans* CH34

Central pathway	Gene	ORF (aa)	CDS	Function	Organism	Id (%)	Cov (%)	UniProtKB Accession (aa)
Catechol (extradiol)	*tomR*	*RMET_RS06580 (614)*	TomR	Transcriptional activator of aromatic catabolism, XylR/NtrC type	*B. vietnamiensis G4*	97	98	A4JW32 (611)
	tomD	*RMET_RS06585 (275)*	TomD	Semialdehyde-2-hydroxymuconate hydrolase (HMSH)	*B. vietnamiensis G4*	100	100	A4JW31 (275)
	tomI	*RMET_RS06645 (63)*	TomI	4-oxalocrotonate tautomerase	*B. vietnamiensis G4*	100	100	A4JW27 (63)
	tomH	*RMET_RS06650 (262)*	TomH	4-oxalocrotonate descarboxylase	*B. vietnamiensis G4*	99	99	A4JW26 (260)
	tomG	*RMET_RS06655 (348)*	TomG	4-hydroxy-2-ketovalerate aldolase	*B. vietnamiensis G4*	99	100	A4JW25 (343)
	tomF	RMET_RS06660 (303)	TomF	Acetaldehyde-CoA dehydrogenase II	*B. vietnamiensis G4*	99	100	A4JW24 (299)
	tomE	*RMET_RS06665 (262)*	TomE	2-hydroxypent-2,4-dienoate hydratase	*B. vietnamiensis G4*	99	100	A4JW23 (258)
	tomC	*RMET_RS06670 (503)*	TomC	Semialdehyde-2-hydroxymuconate dehydrogenase (HMSD)	*B. vietnamiensis G4*	98	100	A4JW22 (493)
	tomB	*RMET_RS06680 (314)*	TomB	Catechol-2,3-dioxygenase (C23O)	*B. vietnamiensis G4*	100	100	A4JW20 (311)
Catechol (intradiol β-ketoadipate)	*catA1*	*RMET_RS08920 (305)*	CatA1	Catechol-1,2-dioxygenase (C12O)	*A. lwoffii* K24	71	84	O33950 (275)
	catD	*RMET_RS25015 (258)*	CatD	3-oxoadipate enol-lactonase	*A. baylyi* ADP1	44	96	P00632 (267)
	catC	RMET_RS25025 (92)	CatC	Muconolactone delta-isomerase	*C. pinatubonensis* JMP134	84	100	P80573 (92)
	catB	*RMET_RS25030 (374)*	CatB	Muconate cycloisomerase	*C. pinatubonensis* JMP134	93	98	P08310 (375)
	catM	*RMET_RS25035 (291)*	CatM	LysR family transcriptional regulator	*P. putida* PSR2000	58	100	P20667 (289)
	benM	*RMET_RS25040 (305)*	BenM	LysR family transcriptional regulator	*A. baylyi* ADP1	48	98	O68014 (304)
	catA2	*RMET_RS25045 (307)*	CatA2	Catechol-1,2-dyoxygenase	*A. lwoffii* K24	65	93	O33948 (275)
Protocatechuate (intradiol β-ketoadipate)	*pcaI*	*RMET_RS19060 (229)*	PcaI	3-oxoacid CoA-transferase subunit A	*P. putida* PRS2000	74	93	Q01103 (231)
	pcaJ	*RMET_RS19065 (215)*	PcaJ	3-oxoacid CoA-transferase subunit B	*P. putida* KT2440	75	96	P0A101 (213)
	pcaF	*RMET_RS19070 (400)*	PcaF	3-oxoadipyl-CoA thiolase	*A. baylyi* ADP1	68	100	Q43974 (401)
	pcaK	*RMET_RS20760 (459)*	PcaK	4-hydroxybenzoate transporter	*P. putida* PRS2000	57	95	Q51955 (448)
	pcaQ	*RMET_RS20765 (317)*	PcaQ	LysR family transcriptional regulator	*E. coli* K-12	42	94	P77171 (307)
	pcaH	*RMET_RS20770 (237)*	PcaH	Protocatechuate 3,4-dioxygenase subunit beta	*B. cepacia* DBO1	63	93	P15110 (235)
	pcaG	*RMET_RS20775 (189)*	PcaG	Protocatechuate 3,4-dioxygenase subunit alpha	*P. putida* KT2440	43	100	P00436 (201)
	pcaB	*RMET_RS20780 (490)*	PcaB	3-carboxy-*cis,cis*-muconate cycloisomerase	*A. baylyi* ADP1	49	96	Q59092 (451)
	pcaL	*RMET_RS20785 (392)*	PcaL	Enol-lactone hydrolase/4-CML decarboxylase	*R. jostii* RHA1	42	95	Q0SH24 (400)
Gallate	*pmdE*	*RMET_RS19250 (341)*	PmdE	4-oxalomesaconate hydratase	*C. testosteroni* BR6020	76	99	Q93PS9 (342)
	galD	*RMET_RS19255 (382)*	GalD	4-oxalomesaconate tautomerase	*P. putida* KT2440	51	99	Q88JY0 (361)
	galR	*RMET_RS19260 (406)*	GalR	LysR family transcriptional regulator	*P. putida* KT2440	43	98	Q88JX7 (397)

	galT	*RMET_RS19265 (441)*	GalT	Aromatic acid/H+ symport family MFS transporter	*P. putida* KT2440	55	97	E8ZB61 (449)
	galA	*RMET_RS19270 (430)*	GalA	Gallate 2,3-dioxygenase	*P. putida* KT2440	64	99	Q88JX5 (420)
	galB	*RMET_RS21720 (245)*	GalB	4-oxalomesaconate hydratase	*P. putida* KT2440	77	100	Q88JX8 (258)
	galC	*RMET_RS21725 (237)*	GalC	4-carboxy-4-hydroxy-2-oxoadipate aldolase	*P. putida* KT2440	60	96	Q88JX9 (238)
Homogentisate	*hmgC*	*RMET_RS01425 (215)*	HmgC	Maleylacetoacetate isomerase	*P. xenovorans* LB400	64	100	Q140J8 (214)
	hmgA	*RMET_RS22545 (442)*	HmgA	Homogentisate 1,2-dioxygenase	*P. xenovorans* LB400	81	98	Q140K0 (439)
	hmgB	*RMET_RS22550 (420)*	HmgB	Fumarylacetoacetase	*P. xenovorans* LB400	65	96	Q144Z1 (419)
Hydroxyquinol	*pnpC*	*RMET_RS25940 (296)*	PnpC	Hydroxyquinol 1,2-dioxygenase	*P. putida* DDL-E4	68	95	C6FI44 (290)
	pnpE	*RMET_RS25945 (355)*	PnpE	Maleylacetate reductase	*P. putida* DDL-E4	73	100	C6FI43 (355)
Benzoyl-CoA	*boxA*	*RMET_RS06160 (415)*	BoxA	Benzoyl-CoA oxygenase component A	*A. evansii* KB740	62	99	Q9AIX6 (414)
	boxB	*RMET_RS06165 (474)*	BoxB	Benzoyl-CoA oxygenase component B	*A. evansii* KB740	72	99	Q9AIX7 (473)
	boxC	*RMET_RS06170 (558)*	BoxC	Benzoyl-CoA-dihydrodiol lyase	*A. evansii* KB740	70	97	Q84HH6 (555)
	boxR	*RMET_RS06175 (316)*	BoxR	Transcriptional regulator	*Azoarcus* sp. CIB	50	91	G8B2G2 (300)
Phenylacetyl-CoA	*paaH*	*RMET_RS04900 (507)*	PaaH	3-hydroxybutyryl-CoA dehydrogenase	*B. cenocepacia* J2315	41	94	B4EL90 (518)
	paaF	*RMET_RS15855 (258)*	PaaF	Enoyl-CoA hydratase	*B. cenocepacia* J2315	84	100	B4E7C0 (258)
	paaG	*RMET_RS15860 (280)*	PaaG	2-(1,2-epoxy-1,2-dihydrophenyl)acetyl-CoA isomerase	*B. cenocepacia* J2315	75	93	B4E7B7 (263)
	paaI	*RMET_RS15865 (146)*	PaaI	Hydroxyphenylacetyl-CoA thioesterase PaaI	*B. cenocepacia* J2315	73	94	B4E7B6 (150)
	paaA	*RMET_RS16925 (335)*	PaaA	1,2-phenylacetyl-CoA epoxidase subunit A	*B. cenocepacia* J2315	81	97	B4E5A2 (332)
	paaB	*RMET_RS16930 (95)*	PaaB	1,2-phenylacetyl-CoA epoxidase subunit B	*B. cenocepacia* J2315	86	98	B4E5A1 (94)
	paaC	*RMET_RS16935 (285)*	PaaC	1,2-phenylacetyl-CoA epoxidase subunit C	*B. cenocepacia* J2315	67	97	B4E5A0 (267)
	paaD	*RMET_RS16940 (202)*	PaaD	Phenylacetate-CoA oxygenase subunit PaaD	*B. cenocepacia* J2315	69	91	B4E599 (184)
	paaE	*RMET_RS16945 (361)*	PaaE	Phenylacetate-CoA oxygenase/reductase subunit PaaE	*B. cenocepacia* J2315	59	99	B4E598 (362)
	paaJ	*RMET_RS19070 (400)*	PaaJ	β-ketoadipyl-CoA thiolase	*B. cenocepacia* J2315	88	100	B4E7B8 (400)
2-aminobenzoyl-CoA	*abmE*	*RMET_RS11025 (137)*	AbmE	Translation inhibitor protein	*A. evansii* KB740	60	96	Q93FB4 (132)
	abmD	*RMET_RS11040 (388)*	AbmD	Acyl-CoA dehydrogenase	*A. evansii* KB740	70	94	Q93FB5 (401)
	abmC	*RMET_RS11045 (283)*	AbmC	Enoyl-CoA hydratase/isomerase	*A. evansii* KB740	70	98	Q93FB6 (281)
	abmB	*RMET_RS11050 (264)*	AbmB	3-Hydroxyacyl-CoA dehydrogenase	*A. evansii* KB740	64	94	Q93FB7 (270)
	abmA	*RMET_RS11055 (791)*	AbmA	2-Aminobenzoyl-CoA monooxygenase/reductase	*A. evansii* KB740	66	99	Q93FB8 (773)
3-hydroxyanthranilate	*onbC*	*RMET_RS26575 (174)*	OnbC	3-hydroxyanthranilate 3,4-dioxygenase	*P. phytofirmans* PsJN	88	100	B2T2S5 (174)
	onbR	*RMET_RS26580 (354)*	OnbR	LysR family transcriptional regulator	*Cupriavidus* sp. ST-14	63	98	A0A0K0PM17 (320)

onbE	*RMET_RS26640 (487)*	OnbE	2-aminomuconic 6-semialdehyde dehydrogenase	*Cupriavidus* sp. ST-14	71	97	A0A0K0PM01 (496)
onbH	*RMET_RS26645 (273)*	OnbH	2-oxopent-4-dienoate hydratase	*Cupriavidus* sp. ST-14	67	90	A0A0K0PLY5 (275)
onbJ	*RMET_RS26650 (314)*	OnbJ	Acetaldehyde dehydrogenase	*Cupriavidus* sp. ST-14	73	94	A0A0K0PN84 (316)
onbI	*RMET_RS26655 (345)*	OnbI	4-hydroxy-2-oxovalerate aldolase	*Cupriavidus* sp. ST-14	79	98	A0A0K0PMH1 (346)
onbG	*RMET_RS26660 (254)*	OnbG	4-oxalocrotonate decarboxylase	*P. fluorescens* KU-7	73	77	Q83V28 (251)
onbF	*RMET_RS26665 (145)*	OnbF	2-aminomuconate deaminase	*P. fluorescens* KU-7	75	98	Q83V27 (143)
onbD	*RMET_RS26670 (333)*	OnbD	2-amino-3-carboxymuconate 6-semialdehyde decarboxylase	*Cupriavidus* sp. ST-14	71	96	A0A0K0PM06 (340)
