# Supplementary material for: Cupriavidus metallidurans CH34 Possesses Aromatic Catabolic Versatility and Degrades Benzene in the Presence of Mercury and Cadmium
Source: Microorganisms. 2022 Feb 21;10(2):484. doi: 10.3390/microorganisms10020484 (PMC8879955; doi:10.3390/microorganisms10020484)
Supplement: Supplementary file 1 [file microorganisms-10-00484-s001.zip › microorganisms-1587111-supplementary/TableS4.pdf]

**Table S4. Predicted aromatic peripheral pathways in *C. metallidurans* CH34**

| Central pathway | Peripheral pathway | Gene | ORF (aa) | CDS | Function | Organism | Id (%) | Cov (%) | UniProtKB Accession (aa) |
|---|---|---|---|---|---|---|---|---|---|
| Catechol | Toluene-Benzene (T2MO) | *tomA0* | RMET_RS06715 (74) | TomA0 | Toluene-benzene-2-monooxygenase, component P0 | *B. vietnamensis* G4 | 99 | 75 | Q9ANX5 (97) |
| | | *tomA1* | RMET_RS06710 (331) | TomA1 | Toluene-benzene-2-monooxygenase, component P1 | *B. vietnamensis* G4 | 99 | 99 | Q9ANX4 (331) |
| | | *tomA2* | RMET_RS06705 (89) | TomA2 | Toluene-benzene-2-monooxygenase, component P2 | *B. vietnamensis* G4 | 97 | 98 | Q9ANX3 (89) |
| | | *tomA3* | RMET_RS06700 (519) | TomA3 | Toluene-benzene-2-monooxygenase, component P3 | *B. vietnamensis* G4 | 99 | 100 | Q9ANX2 (519) |
| | | *tomA4* | RMET_RS06695 (118) | TomA4 | Toluene-benzene-2-monooxygenase, component P4 | *B. vietnamensis* G4 | 100 | 100 | Q9ANX1 (118) |
| | | *tomA5* | RMET_RS06690 (354) | TomA5 | Toluene-benzene-2-monooxygenase, component P5 | *B. vietnamensis* G4 | 98 | 100 | Q9ANX0 (354) |
| | | *fer2* | RMET_RS06685 (118) | Fer2 | Ferredoxin | *Pseudomonas* sp. CF600 | 46 | 68 | P23103 (112) |
| | Toluene (TMO) | *tmoA* | RMET_RS06640 (500) | TmoA | Toluene-4-monooxygenase system protein A | *P. mendocina* KR1 | 69 | 69 | Q00456 (500) |
| | | *tmoB* | RMET_RS06635 (88) | TmoB | Toluene-4-monooxygenase system protein B | *P. mendocina* KR1 | 49 | 95 | Q00457 (84) |
| | | *tmoC* | RMET_RS06630 (111) | TmoC | Toluene-4-monooxygenase system protein C | *P. mendocina* KR1 | 53 | 100 | Q00458 (112) |
| | | *tmoD* | RMET_RS06625 (104) | TmoD | Toluene-4-monooxygenase system protein D | *P. mendocina* KR1 | 60 | 91 | Q00459 (103) |
| | | *tmoE* | RMET_RS06620 (328) | TmoE | Toluene-4-monooxygenase system protein E | *P. mendocina* KR1 | 60 | 91 | Q00460 (327) |
| | | *tmoF* | RMET_RS06615 (340) | TmoF | Ferredoxin--NAD(+) reductase | *P. mendocina* KR1 | 43 | 99 | Q8GI14 (329) |
| | | *tmoX* | RMET_RS06590 (464) | TmoX | Aromatic hydrocarbon TbuX/Fad membrane transport protein | *P. mendocina* KR1 | 47 | 87 | Q8KT43 (456) |
| | Phenol (PH) | *phyR* | RMET_RS08955 (542) | PhyR | Transcriptional activator of aromatic catabolism | *Ralstonia* sp. KN1 | 52 | 98 | Q9RAG0 (446) |
| | | *phyZ* | RMET_RS31735 (65) | PhyZ | Phenol hydroxylase P0 protein | *Ralstonia* sp. KN1 | 46 | 98 | P19729 (72) |
| | | *phyA* | RMET_RS08945 (331) | PhyA | Phenol hydroxylase P1 protein | *Ralstonia* sp. KN1 | 54 | 99 | Q9RAF8 (331) |
| | | *phyB* | RMET_RS08940 (90) | PhyB | Phenol hydroxylase P2 protein | *Ralstonia* sp. KN1 | 62 | 96 | Q9RAF7 (90) |
| | | *phyC* | RMET_RS08935 (515) | PhyC | Phenol hydroxylase P3 protein | *Ralstonia* sp. KN1 | 74 | 96 | Q9RAF6 (504) |
| | | *phyD* | RMET_RS08930 (120) | PhyD | Phenol hydroxylase P4 protein | *Ralstonia* sp. KN1 | 55 | 92 | Q9RAF5 (119) |
| | | *phyE* | RMET_RS08925 (356) | PhyE | Phenol hydroxylase P5 protein | *Ralstonia* sp. KN1 | 62 | 100 | Q9RAF4 (356) |
| | Benzoate (BDO) | *benA* | RMET_RS25050 (464) | BenA | Benzoate 1,2-dioxygenase subunit alpha | *A. baylyi* ADP1 | 73 | 96 | P07769 (461) |
| | | *benB* | RMET_RS25055 (164) | BenB | Benzoate 1,2-dioxygenase subunit beta | *A. baylyi* ADP1 | 70 | 98 | P07770 (169) |
| | | *benC* | RMET_RS25060 (339) | BenC | Benzoate 1,2-dioxygenase electron transfer component | *A. baylyi* ADP1 | 65 | 98 | P07771 (348) |
| | | *benD* | RMET_RS25065 (264) | BenD | DHB dehydrogenase | *A. baylyi* ADP1 | 62 | 100 | P07772 (261) |
| Protocatechuate | Ferulic acid | *fcs* | RMET_RS25745 (631) | Fcs | Feruloyl-CoA synthase | *S. stellata* E-37 | 39 | 86 | A3K467 (610) |
| | Feruloyl-CoA | *ech* | RMET_RS11875 (289) | Ech | Crotonase/enoyl-CoA hydratase | *S. stellata* E-37 | 45 | 96 | A3K472 (261) |
| | Conyferal alcohol | *calA* | RMET_RS05500 (342) | CalA | Coniferyl alcohol dehydrogenase | *P. putida* KT2440 | 43 | 98 | Q88G86 (336) |
| | Conyferal aldehyde | *calB* | RMET_RS00805 (476) | CalB | Coniferyl aldehyde dehydrogenase | *P. putida* KT2440 | 52 | 99 | Q88CR0 (476) |
| | Vanillin | *vdh* | RMET_RS25355 (482) | Vdh | Vanillin dehydrogenase | *Pseudomonas* sp. HR199 | 37 | 99 | O05619 (481) |
| | Vanillate | *ivaA* | RMET_RS28335 (347) | IvaA | Isovanillate *O*-demethylase | *C. testosteroni* BR6020 | 41 | 97 | Q2KQ79 (347) |
| | | *ivaB* | RMET_RS28190 (331) | IvaB | Isovanillate *O*-demethylase oxidoreductase | *C. testosteroni* BR6020 | 45 | 97 | Q2KQ78 (331) |
| | 4-hydroxybenzoate | *pobA1* | RMET_RS20795 (398) | PobA1 | 4-hydroxybenzoate-3-monooxygenase | *A. baylyi* ADP1 | 64 | 98 | Q03298 (404) |
| | | *pobA2* | RMET_RS28050 (392) | PobA2 | 4-hydroxybenzoate-3-monooxygenase | *Paenibacillus* sp. JJ-1b | 65 | 98 | C4TP09 (394) |
| Homogentisate | Tyrosine | *tyrB1* | RMET_RS05120 (398) | TyrB1 | Tyrosine aminotransferase | *P. aeruginosa* PAO1 | 75 | 99 | P72173 (398) |

| | | | | | | | | | |
|---|---|---|---|---|---|---|---|---|---|
| Homogentisate | | *tyrB2* | RMET_RS25280 (398) | TyrB2 | Tyrosine aminotransferase | *K. pneumoniae* ATCC 13883 | 57 | 99 | O85746 (397) |
| | Phenylalanine | *phhA* | RMET_RS17750 (310) | PhhA | Phenylalanine 4-monooxygenase | *R. solaneacearum* GMI1000 | 80 | 99 | Q8XU39 (313) |
| | | *phhB* | RMET_RS17755 (99) | PhhB | 4α-carbinolamine dehydratase | *C. pinatubonensis* JMP134 | 82 | 97 | Q46VT8 (100) |
| | 4-HPP | *hppD* | RMET_RS25270 (357) | HppD | 4-hydroxyphenylpyruvate dioxygenase | *Pseudomonas* sp. P.J. 874 | 78 | 99 | P80064 (357) |
| | 2-HPA | *ohpA* | RMET_RS25305 (780) | OhpA | Cytochrome P450 CYP116B (2-HPA hydroxylase) | *C. pinatubonensis* JMP134 | | | AAZ64624 (783) |
| Phenylacetyl-CoA | Phenylacetate | *paaK* | RMET_RS15870 (434) | PaaK | Phenylacetate-CoA ligase | *B. cenocepacia* J2315 | 79 | 97 | B4E7B5 (432) |
| Benzoyl-CoA | Benzoate (Bcl) | *bclA* | RMET_RS06180 (549) | BclA | Benzoate--CoA ligase | *T. aromatica* K172 | 62 | 93 | Q8GQN9 (527) |
| Aminobenzoyl-CoA | Tryptophan | *kynB* | RMET_RS13315 (-) | KynB | Arylformamidase | PSEUDOGEN | - | - | - |
| | | *kynU1* | RMET_RS13320 (418) | KynU1 | Kynureninase | *P. fluorescens* | 72 | 99 | P83788 (414) |
| | | *kynA* | RMET_RS13325 (299) | KynA | Tryptophan 2,3-dioxygenase | *C. necator* H16 | 88 | 100 | Q0K7X7 (294) |
| | | *kynU2* | RMET_RS26570 (409) | KynU2 | Kynureninase | *P. fluorescens* | 53 | 100 | P83788 (416) |
| | 2-aminobenzoate | *abmG* | RMET_RS11035 (553) | AbmG | 2-aminobenzoate-CoA ligase | *A. evansii* KB740 | 58 | 99 | Q93FC0 (542) |
