# Supplementary material for: Cupriavidus metallidurans CH34 Possesses Aromatic Catabolic Versatility and Degrades Benzene in the Presence of Mercury and Cadmium
Source: Microorganisms. 2022 Feb 21;10(2):484. doi: 10.3390/microorganisms10020484 (PMC8879955; doi:10.3390/microorganisms10020484)
Supplement: Supplementary file 1 [file microorganisms-10-00484-s001.zip › microorganisms-1587111-supplementary/TableS5.pdf]

Table S5. Statistic analyses of mercury and cadmium effects on benzene degradation and intermediates production by *C. metallidurans* CH34 and *P. putida* F1.

	***C. metallidurans* CH34**						***P. putida* F1**			
	**Hg 32.5 μM**			**Cd 200 μM**			**Hg 32.5 μM**		**Cd 200 μM**	
**Time (h)**	**% Residual benzene**	**Phenol (mM)**	**2-HMS (μM)**	**% Residual benzene**	**Phenol (mM)**	**2-HMS (μM)**	**% Residual benzene**	**2-HMS (μM)**	**% Residual benzene**	**2-HMS (μM)**
0	n/a	ns	ns	n/a	ns	ns	n/a	ns	n/a	ns
3	ns	ns	*	ns	*	**	***	***	ns	ns
6	*	**	**	*	**	***	***	***	ns	***
9	ns	ns	ns	a	ns	ns	***	***	ns	*
12	ns	ns	*	ns	ns	ns	***	**	ns	ns

Significance results correspond to *p* values obtained from the t-test using control values as reference. n/a: not applicable, ns: $p>0.05$, *: $p\leq0.05$, **: $p\leq0.01$, ***: $p\leq0.001$.
